# Supplementary material for: Near-Infrared Spectroscopy Monitoring in Cardiac and Noncardiac Surgery: Pairwise and Network Meta-Analyses
Source: J Clin Med. 2019 Dec 14;8(12):2208. doi: 10.3390/jcm8122208 (PMC6947303; doi:10.3390/jcm8122208)
Supplement: Supplementary file 1 [file jcm-08-02208-s001.pdf]

Supplementary Materials

Near-Infrared Spectroscopy Monitoring in Cardiac and Noncardiac Surgery: Pairwise and Network Meta-Analyses

Christian Ortega-Loubon, Francisco Herrera-Gómez, Carolina Bernuy-Guevara, Pablo Jorge-Monjas, Carlos Ochoa-Sangrador, Juan Bustamante-Munguira, Eduardo Tamayo and F. Javier Álvarez.

Material S1. Supplementary methods and search strategy
Table S1. Participants, interventions, comparators, and outcomes in the included trials.
Table S2. Participants, interventions, comparators, and outcomes in trials eligible but not providing numerical data for the analysis.
Table S3. Assessing risk of bias in trials eligible.
Figure S1. The effect of NIRS monitoring on post-operative death.
Figure S2. The effect of NIRS monitoring on post-operative AKI.
Figure S3. The effect of NIRS monitoring on MACEs.
Figure S4. The effect of NIRS monitoring on bleeding/need for transfusions.
Figure S5. The effect of NIRS monitoring on morbidity combining POCD/POD, AKI and MACEs.
Table S4. The ranking of SUCRA assessable NIRS target values for three outcomes.
Figure S6. Network forests plot of the random effects (vague priors) for the SUCRA assessable NIRS target values corresponding to (A) post-operative death, (B) post-operative AKI, and (C) MACEs.
Figure S7. Inconsistency plot of the random effects for the SUCRA assessable NIRS target values corresponding to (A) post-operative death, (B) post-operative AKI, and (C) MACEs.

Material S1
Supplementary methods and search strategy

Full details on the methods followed are available to the reader from the online access link to the registered systematic review protocol in the International prospective register of systematic reviews PROSPERO:
Francisco Herrera-Gómez, Christian Ortega-Loubon, Coralina Bernuy-Guevara, Carlos Ochoa-Sangrador, Juan Bustamante-Munguira, Pablo Jorge-Monjas, Eduardo Tamayo, F. Javier Álvarez. Perioperative targets near-infrared spectroscopy values for cardiac and non-cardiac surgery patients: pairwise and network meta-analyses. PROSPERO 2019 CRD42019131417. Available from:
http://www.crd.york.ac.uk/PROSPERO/display_record.php?ID=CRD42019131417

The published and unpublished literature sources interrogated, and the strategy designed and constructed at Pharmacological Big Data Laboratory, Pharmacology and Therapeutics, Faculty of Medicine, University of Valladolid, Spain, on the basis of search terms referred to the participants/population, intervention and study design are also available to the reader into the following panel (search results and formula):

**PubMed**
319221: "Thoracic Surgical Procedures"[MeSH Terms]<br>369697: "Cardiovascular Surgical Procedures"[MeSH Terms]<br>207356: "Cardiac Surgical Procedures"[MeSH Terms]<br>89485: "Myocardial Revascularization"[MeSH Terms]<br>428642: (("bypass"[Text Word]) OR "graft"[Text Word]) OR "grafting"[Text Word]<br>357261: (("valve"[Text Word]) OR "mitral"[Text Word]) OR "aortic"[Text Word]<br>66910: "Extracorporeal Circulation"[MeSH Terms] OR "Cardiopulmonary Bypass"[MeSH Terms]<br>45180: "extracorporeal"[Text Word]<br>2251: "intra aortic balloon pump"[Text Word]<br>84214: "Anastomosis, Surgical"[MeSH Terms]<br>75621: "anastomosis"[Text Word]<br>350277: "Digestive System Surgical Procedures"[MeSH Terms]<br>12718: "Elective Surgical Procedures"[MeSH Terms]<br>18432: "Laparotomy"[MeSH Terms]<br>21339: "Splenectomy"[MeSH Terms]<br>792: "Metastasectomy"[MeSH Terms]<br>712919: ((("gastric"[Text Word]) OR "biliary"[Text Word]) OR "pancreatic"[Text Word]) OR "colorectal"[Text Word]<br>29203: "splenectomy"[Text Word]<br>269744: "resection"[Text Word]<br>184638: "Neurosurgical Procedures"[MeSH Terms]<br>25363: "Pneumonectomy"[MeSH Terms]<br>82331: ((("lobectomy"[Text Word]) OR "craniotomy"[Text Word]) OR "craniectomy"[Text Word]) OR "decompression"[Text Word]<br>29560: ("pneumonectomy"[Text Word]) OR "segmentectomy"[Text Word]<br>289291: Orthopedic Procedures"[MeSH Terms]

281836: ("knee"[Text Word]) OR "hip"[Text Word]<br>32954: ("arthroscopy"[Text Word]) OR "arthroscopic"[Text Word]<br>201582: "Organ Transplantation"[MeSH Terms]<br>711049: (("transplantation"[Text Word]) OR "transplanted"[Text Word]) OR "transplant"[Text Word]<br>12310: "Spectroscopy, Near-Infrared"[MeSH Terms]<br>17817: ("near infrared spectroscopy"[Text Word]) OR "nirs"[Text Word]<br>327739: "Clinical Trials as Topic"[MeSH Terms]<br>132621: "Controlled Clinical Trials as Topic"[MeSH Terms]<br>127557: "Randomized Controlled Trials as Topic"[MeSH Terms]<br>99572: "Random Allocation"[MeSH Terms]<br>1251537: ((("random"[Text Word]) OR "randomization"[Text Word]) OR "randomized"[Text Word]) OR "randomly"[Text Word]<br>178207: ("Double-Blind Method"[MeSH Terms]) OR "Single-Blind Method"[MeSH Terms]<br>322691: (("blind"[Text Word]) OR "blinded"[Text Word]) OR "masked"[Text Word]<br>34399: "Placebos"[MeSH Terms]<br>205494: "placebo"[Text Word]
**Ovid**<br>Journals@Ovid (Sumarios y Resúmenes todas las Revistas Ovid), Revistas Subscritas a Texto Completo por Sacyl, Ovid MEDLINE(R) and Epub Ahead of Print, In-Process & Other Non-Indexed Citations, Daily and Versions(R) 1946 to May 25, 2019
319194: exp Thoracic Surgical Procedures/<br>369653: exp Cardiovascular Surgical Procedures/<br>207341: exp Cardiac Surgical Procedures/<br>89481: exp Myocardial Revascularization/<br>963924: bypass.tw. or graft$.tw.<br>675360: valve.tw. or mitral.tw. or aortic.tw.<br>66908: exp Extracorporeal Circulation/ or exp Cardiopulmonary Bypass/<br>88362: extracorporeal.tw.<br>9579: intra aortic balloon pump.tw.<br>84196: exp Anastomosis, Surgical/<br>142202: anastomosis.tw.<br>350226: exp Digestive System Surgical Procedures/<br>12714: exp Elective Surgical Procedures/<br>18429: exp Laparotomy/<br>21336: exp Splenectomy/<br>792: exp Metastasectomy/<br>1159695: gastric.tw. or biliary.tw. or pancreatic.tw. or colorectal.tw.<br>43414: splenectomy.tw.<br>608657: resection.tw.<br>184569: exp Neurosurgical Procedures/<br>25361: exp Pneumonectomy/<br>180563: lobectomy.tw. or craniotomy.tw. or craniectomy.tw. or decompression.tw.<br>23181: pneumonectomy.tw. or segmentectomy.tw.<br>289209: exp Orthopedic Procedures/<br>543893: knee.tw. or hip.tw.

62126: arthroscop$.tw.
201533: exp Organ Transplantation/
1074634: transplant$.tw.
12306: exp Spectroscopy, Near-Infrared/
23308: near infrared spectroscopy.tw. or nirs.tw.
327595: exp Clinical Trials as Topic/
132514: exp Controlled Clinical Trials as Topic/
127452: exp Randomized Controlled Trials as Topic/
99543: exp Random Allocation/
2905965: random$.tw.
178161: exp Double-Blind Method/ or exp Dingle-Blind Method/
1260536: blind$.tw. or mask$.tw.
34396: exp Placebos/
588826: placebo$.tw.

Elsevier's Scopus

4662: KEY ("thorax surgery") AND NOT INDEX (medline)
2701: KEY("cardiovascular surgery") AND NOT INDEX(medline)
14586: KEY("heart surgery") AND NOT INDEX(medline)
5167: KEY("heart muscle revascularization") AND NOT INDEX(medline)
236867: (TITLE-ABS-KEY(bypass) OR TITLE-ABS-KEY(graft*)) AND NOT INDEX(medline)
198251: (TITLE-ABS-KEY(valve) OR TITLE-ABS-KEY(mitral) OR TITLE-ABS-KEY(aortic)) AND NOT INDEX(medline)
10552: (KEY("extracorporeal circulation") OR KEY("cardiopulmonary bypass")) AND NOT INDEX(medline)
14882: TITLE-ABS-KEY(extracorporeal) AND NOT INDEX(medline)
650: TITLE-ABS-KEY("intra aortic balloon pump") AND NOT INDEX(medline).
16785: KEY(anastomosis) AND NOT INDEX(medline)
25639: TITLE-ABS-KEY(anastomosis) AND NOT INDEX(medline)
5644: KEY("abdominal surgery") AND NOT INDEX(medline)
6294: KEY("elective surgery") AND NOT INDEX(medline)
15483: KEY(laparotomy) AND NOT INDEX(medline)
5384: KEY(splenectomy) AND NOT INDEX(medline)
564: KEY("metastasis resection") AND NOT INDEX(medline)
195529: (TITLE-ABS-KEY(gastric) OR TITLE-ABS-KEY(biliary) OR TITLE-ABS-KEY(pancreatic) OR TITLE-ABS-KEY(colorectal)) AND NOT INDEX(medline)
7523: TITLE-ABS-KEY(splenectomy) AND NOT INDEX(medline)
101314: TITLE-ABS-KEY(resection) AND NOT INDEX(medline)
15474: KEY(neurosurgery) AND NOT INDEX(medline)
5077: KEY("lung resection") AND NOT INDEX(medline)
43421: (TITLE-ABS-KEY(lobectomy) OR TITLE-ABS-KEY(craniotomy) OR TITLE-ABS-KEY(craniectomy) OR TITLE-ABS-KEY(decompression)) AND NOT INDEX(medline)
3561: (TITLE-ABS-KEY(pneumonectomy) OR TITLE-ABS-KEY(segmentectomy)) AND NOT INDEX(medline)
7945: KEY("orthopedic surgery") AND NOT INDEX(medline)
127589: (TITLE-ABS-KEY(knee) OR TITLE-ABS-KEY(hip)) AND NOT INDEX(medline)
14910: TITLE-ABS-KEY(arthroscop*) AND NOT INDEX(medline)

5900: KEY("organ transplantation") AND NOT INDEX(medline)
168394: TITLE-ABS-KEY(transplant*) AND NOT INDEX(medline)
13570: KEY("near infrared spectroscopy") AND NOT INDEX(medline)
5567: TITLE-ABS-KEY(nirs) AND NOT INDEX(medline)
26127: KEY("clinical trial (topic)") AND NOT INDEX(medline)
1736: KEY("controlled clinical trial (topic)") AND NOT INDEX(medline)
33114: KEY("randomized controlled trial (topic)") AND NOT INDEX(medline)
13683: KEY(randomization) AND NOT INDEX(medline)
1211201: TITLE-ABS-KEY(random*) AND NOT INDEX(medline)
30368: (KEY("double blind procedure") OR KEY("single blind procedure")) AND NOT INDEX(medline)
303111: (TITLE-ABS-KEY(blind*) OR TITLE-ABS-KEY(mask*)) AND NOT INDEX(medline)
64114: KEY(placebo) AND NOT INDEX(medline)
94263: TITLE-ABS-KEY(placebo) AND NOT INDEX(medline)

Web of Science
Web of Science Core Collection, Current Contents Connect, Derwent Innovations Index, KCI-Korean Journal Database, MEDLINE®, Russian Science Citation Index, SciELO Citation Index

864772: TOPIC: (bypass) OR TOPIC: (graft*)
1205349: TOPIC: (valve) OR TOPIC: (mitral) OR TOPIC: (aortic)
64984: TOPIC: (extracorporeal)
3190: TOPIC: ("intra aortic balloon pump")
112472: TOPIC: (anastomosis)
1164321: TOPIC: (gastric) OR TOPIC: (biliary) OR TOPIC: (pancreatic) OR TOPIC: (colorectal)
34282: TOPIC: (splenectomy)
397960: TOPIC: (resection)
133776: TOPIC: (lobectomy) OR TOPIC: (craniotomy) OR TOPIC: (craniectomy) OR TOPIC: (decompression)
32914: TOPIC: (pneumonectomy) OR TOPIC: (segmentectomy)
459043: TOPIC: (knee) OR TOPIC: (hip)
45282: TOPIC: (arthroscop*)
1208997: TOPIC: (transplant*)
35045: TOPIC: ("near infrared spectroscopy") OR TOPIC: (nirs)
2457139: TOPIC: (random*)
1016208: TOPIC: (blind*) OR TOPIC: (mask*)
315421: TOPIC: (placebo*)

Cochrane Controlled Register of Trials (CENTRAL)

14931: MeSH descriptor: [Thoracic Surgical Procedures] explode all trees
18619: MeSH descriptor: [Cardiovascular Surgical Procedures] explode all trees
12443: MeSH descriptor: [Cardiac Surgical Procedures] explode all trees
8987: MeSH descriptor: [Myocardial Revascularization] explode all trees
36950: (bypass):ti,ab,kw OR (graft):ti,ab,kw OR (grafting):ti,ab,kw
14535: (valve):ti,ab,kw OR (mitral):ti,ab,kw OR (aortic):ti,ab,kw

MeSH descriptor: [Extracorporeal Circulation] explode all trees (a)

MeSH descriptor: [Cardiopulmonary Bypass] explode all trees (b)<br>3890: a OR b<br><br>3987: (extracorporeal):ti,ab,kw<br>279: ("intra-aortic balloon pump"):ti,ab,kw<br>2102: MeSH descriptor: [Anastomosis, Surgical] explode all trees<br>3967: (anastomosis):ti,ab,kw<br>13022: MeSH descriptor: [Digestive System Surgical Procedures] explode all trees<br>1845: MeSH descriptor: [Elective Surgical Procedures] explode all trees<br>708: MeSH descriptor: [Laparotomy] explode all trees<br>175: MeSH descriptor: [Splenectomy] explode all trees<br>11: MeSH descriptor: [Metastasectomy] explode all trees<br>52804: (gastric):ti,ab,kw OR (biliary):ti,ab,kw OR (pancreatic):ti,ab,kw OR (colorectal):ti,ab,kw<br>658: (splenectomy):ti,ab,kw<br>20371: (resection):ti,ab,kw<br>5605: MeSH descriptor: [Neurosurgical Procedures] explode all trees<br>506: MeSH descriptor: [Pneumonectomy] explode all trees<br>5226: (lobectomy):ti,ab,kw OR (craniotomy):ti,ab,kw OR (craniectomy):ti,ab,kw OR (decompression):ti,ab,kw<br>872: (pneumonectomy):ti,ab,kw OR (segmentectomy):ti,ab,kw<br>11550: MeSH descriptor: [Orthopedic Procedures] explode all trees<br>41269: (knee):ti,ab,kw OR (hip):ti,ab,kw<br>3974: (arthroscopy):ti,ab,kw OR (arthroscopic):ti,ab,kw<br>5434: MeSH descriptor: [Organ Transplantation] explode all trees<br>35657: (transplantation):ti,ab,kw OR (transplanted):ti,ab,kw OR (transplant):ti,ab,kw<br>299: MeSH descriptor: [Spectroscopy, Near-Infrared] explode all trees<br>1282: ("near-infrared spectroscopy"):ti,ab,kw OR (nirs):ti,ab,kw<br>40986: MeSH descriptor: [Clinical Trials as Topic] explode all trees<br>7764: MeSH descriptor: [Controlled Clinical Trials as Topic] explode all trees<br>7674: MeSH descriptor: [Randomized Controlled Trials as Topic] explode all trees<br>20584: MeSH descriptor: [Random Allocation] explode all trees<br>899229: (random):ti,ab,kw OR (randomization):ti,ab,kw OR (randomized):ti,ab,kw OR (randomly):ti,ab,kw<br><br>MeSH descriptor: [Double-Blind Method] explode all trees (c)<br>MeSH descriptor: [Single-Blind Method] explode all trees (d)<br>150464: c or d<br><br>345626: (blind):ti,ab,kw OR (blinded):ti,ab,kw OR (masked):ti,ab,kw<br>23507: MeSH descriptor: [Placebos] explode all trees<br>270449: (placebo):ti,ab,kw
**Study registries**<br>ClinicalTrials.gov, the EU Clinical Trials Register, the United Kingdoms' ISRCTN registry
1087: bypass

3044: graft
Also searched for Grafting in ClinicalTrials.gov

1876: valve
364: mitral
1825: aortic
Also searched for Aorta in ClinicalTrials.gov

522: extracorporeal
15: "intra aortic balloon pump"
Also searched for Balloon pump in ClinicalTrials.gov

380: anastomosis
Also searched for Anastomotic in ClinicalTrials.gov

4431: gastric
Also searched for Stomach and Gastro in ClinicalTrials.gov
1351: biliary
4720: pancreatic
Also searched for Pancreas in ClinicalTrials.gov

5555: colorectal
44: splenectomy
3183: resection
Also searched for Removal, Excisions, and Resected in ClinicalTrials.gov

124: lobectomy
126: craniotomy
24: craniectomy
220: decompression
24: pneumonectomy
27: segmentectomy
4133: knee
2154: hip
233: arthroscopy
304: arthroscopic
270: "near infrared spectroscopy"
222: nirs
60663: random or randomization
Also searched for Randomized in ClinicalTrials.gov

16: randomly
27858: blind
3899: blinded
1444: masked
Also searched for Mask in ClinicalTrials.gov

24503: placebo

Also searched for Placebo-controlled in ClinicalTrials.gov
**DART Europe E-Theses Portal**
1164: bypass<br>1515: graft<br>943: valve<br>221: mitral<br>1113: aortic<br>181: extracorporeal<br>8: "intra aortic balloon pump"<br>220: anastomosis<br>1073: gastric<br>328: biliary<br>1287: pancreatic<br>1630: colorectal<br>47: splenectomy<br>876: resection<br>23: lobectomy<br>20: craniotomy<br>7: craniectomy<br>202: decompression<br>15: pneumonectomy<br>2: segmentectomy<br>1103: knee<br>0: hip<br>66: arthroscopy<br>61: arthroscopic<br>229: "near infrared spectroscopy"<br>188: nirs<br>9304: random or randomization or randomized<br>2278: randomly<br>1409: blind<br>282: blinded<br>321: masked<br>1320: placebo
**Open Access Theses and Dissertations (OATD)**
5277: bypass<br>5617: graft<br>5673: valve<br>920: mitral<br>4189: aortic<br>562: extracorporeal<br>24: "intra aortic balloon pump"<br>723: anastomosis<br>4896: gastric<br>1013: biliary<br>5338: pancreatic<br>5732: colorectal

156: splenectomy<br>2815: resection<br>116: lobectomy<br>120: craniotomy<br>36: craniectomy<br>891: decompression<br>43: pneumonectomy<br>9: segmentectomy<br>7134: knee<br>7969: hip<br>226: arthroscopy<br>258: arthroscopic<br>1178: "near infrared spectroscopy"<br>777: nirs<br>72978: random or randomization or randomized<br>29399: randomly<br>9274: blind<br>1761: blinded<br>2293: masked<br>6568: placebo
**Meeting abstracts archives**<br>The World Heart and Cardiothoracic Surgery Conference to 2018, World Congress of Surgery to 2018, and World Congress of Anaesthesiologists to 2018.
233: manual searches.

Table S1. Participants, interventions, comparators, and outcomes in the included trials.

Trial Details	Design	Follow-up (days)	Participants/Population Characteristics	Interventions (*n*)	Comparators (*n*)	Outcomes	Cointerventions
**Colak et al., 2015** [1] NCT00917124 Croatia	RCT	7	≥65 years/male/DM (%): 41.0/78.0/34.2 Operation: CABG with CPB. NIRS: INVOS system.	bSO2 >80%[$] (94)	No NIRS monitoring (96)	Neurocognitive impairment (MMSE, CTT1, GP), MACE, BPN, HD, PMV, ICU/hospital LOS.	None
**NORMOSAT** [2] NCT01432184 Canada	RCT	30	≥65 years/male/DM (%): 66.0/72.1/29.4 Operation: CABG + VR/r, multiple VR/r, Redo. NIRS: INVOS and non-INVOS systems.	Non-drop bSO_2 >20%[$] (102)	No NIRS monitoring objectives (99)	Delirium[€], death, MACE, arrhythmia, BPN, AKI, PMV, ICU/hospital LOS, readmission.	None
**Kara et al., 2016** [3] Turkey	RCT	7-10	≥65 years/male/DM (%): 36.5/78.5/30.4 Operation: CABG with CPB. NIRS: INVOS system.	Non-drop bSO_2 >20%[$] (43)	No NIRS monitoring (36)	Neurocognitive impairment (MoCa), ICU/hospital LOS.	None
**Mohandas et al., 2013** [4] India	RCT	90	≥65 years/male/DM (%): 13.5/58.0/0.0 Operation: CABG with CPB. NIRS: non-INVOS system.	bSO2 >85%[$] (50)	No NIRS monitoring objectives (50)	Neurocognitive impairment (MMSE, ASEM).	None
**Slater et al., 2009** [5] USA	RCT	90	≥65 years/male/DM (%): 38.0/84.0/35.0 Operation: CABG with CPB. NIRS: INVOS system.	Non-drop bSO_2 >20%[$] (125)	No NIRS monitoring objectives (115)	Neurocognitive impairment[€], delirium (DRS), hospital LOS.	None
**Murkin et al., 2007** [6] Canada	RCT	30	≥65 years/male/DM (%): 24.0/88.0/21.0 Operation: CABG with CPB. NIRS: INVOS system.	bSO2 >75%[$] (100)	No NIRS monitoring objectives (100)	Death, MACE, arrhythmia, HD, PMV, ICU/hospital LOS, readmission.	None
**Vretzakis et al., 2013** [7] NCT00879463 Greece	RCT	30	≥65 years/male/DM (%): 27.7/83.0/23.4 Operation: CABG with CPB. NIRS: INVOS system.	Non-drop bSO_2 >20%[$] (75)	No NIRS monitoring objectives (75)	Neurocognitive impairment[€], death, MACE, HD, PMV, ICU/hospital LOS.	NIRS-guided transfusions.

**PASPORT** [8]<br>ISRCTN 23557269<br>UK	RCT	90	≥65 years/male/DM (%): 19.0/68.0/8.8<br>Operation: CABG with CPB.<br>NIRS: INVOS system.	bSO2 >70%[$] (98)	No NIRS monitoring (106)	Neurocognitive impairment[€], death, MACE, BPN, AKI, biomarkers[£].	hematocrit-guided transfusions.
**Cowie et al., 2014** [9]<br>Australia	RCT	30	≥65 years/male/DM (%): 40.0/50.0/10.0<br>Operation: colorectal, orthopedic.<br>NIRS: INVOS system.	bSO2 >75%[$] (20)	No NIRS monitoring (20)	Death, MACE, AKI, hospital LOS, readmission.	None
**Ballard et al., 2012** [10]<br>ISRCTN39503939<br>UK	RCT	364	≥65 years/male/DM (%): 100.0/38.5/10.0<br>Operation: abdominal, orthopedic.<br>NIRS: non-INVOS system.	Non-drop bSO_2 >15%[$] (22)	No NIRS monitoring (29)	Neurocognitive impairment (MMSE), biomarkers[£].	None
**Casati et al., 2007** [11]<br>USA	RCT	7-20	≥65 years/male/DM (%): 100.0/38.5/0.0<br>Operation: abdominal.<br>NIRS: INVOS system.	bSO2 >75%[$] (56)	No NIRS monitoring objectives (66)	Neurocognitive impairment (MMSE), ICU/hospital LOS.	None
**Cox et al., 2018** [12]<br>Netherlands	RCT	42	≥65 years/male/DM (%): 40.0/65.5/0.0<br>Operation: Orthopedic.<br>NIRS: non-INVOS system.	Non-drop bSO_2 >20%[$] (20)	No NIRS monitoring objectives (20)	Neurocognitive impairment (MoCa).	None

[$] Target values were set on the basis of baseline bSO_2 measurement. [€] standard scale use was not informed or not used. [£] serum S100B and IL-6 and urinary NGAL protein were dosed for detecting, respectively, brain injury, systemic inflammation and AKI in PASPORT study; only serum S100B was dosed in the study of Ballard et al., 2012 (reference 10). Abbreviations: AKI, acute kidney injury; ASEM, antisaccadic eye movement test; BPN, blood product need; bSO_2, brain oxygen saturation; CABG, coronary artery bypass grafting; CPB, cardiopulmonary bypass; DM, diabetes mellitus; DRS, Delirium Rating Scale; GP, Grooved-Pegboard test; GTT1, Color Trail Test 1; HD, hemodialysis; ICU, intensive care unit; INVOS, IN Vivo Optical Spectroscopy; LOS, length of stay; MACE, major adverse cardiovascular event; MMSE, Mini-Mental State Examination; MoCa, Montreal Cognitive Assessment test; NIRS, near-infrared spectroscopy; NORMOSAT, NORMal cerebral Oxygen SATuration; PASPORT, PAtient-Specific cerebral oxygentation monitoring as Part Of an algorithm to Reduce Transfusion during heart valve surgery: a randomised controlled trial; PMV, prolonged mechanical ventilation; RCT, randomized controlled trial; redo; re-operative cardiac surgery; VR/r, valve replacement or repair.

Table S2. Participants, interventions, comparators, and outcomes in trials eligible but not providing numerical data for the analysis.

Trial Details	Design	Follow-up (days)	Participants/Population Characteristics	Interventions (*n*)	Comparators (*n*)	Outcomes	Cointerventions
**Deschamps et al., 2013** [13] Canada	RCT	7	≥65 years/male/DM (%): 66.0/72.1/29.4 Operation: CABG + VR/r, multiple VR/r, Redo. NIRS: INVOS and non-INVOS systems.	Non-drop bSO_2 >20%[$] (23)	No NIRS monitoring objectives (25)	BPN, PMV, ICU/hospital LOS, readmission.	None
**Harilall et al., 2014** [14] South Africa	RCT	30	≥65 years/male/DM (%): 51.0/70.0/19.0 Operation: CABG with CPB. NIRS: INVOS system.	bSO2 >75%[$] (20)	No NIRS monitoring (20)	MAP, temperature, pCO_2, hematocrit, biomarkers[¥].	None
**Trafidło et al., 2015** [15] Poland	RCT	42	≥65 years/male/DM (%): 20.0/70.0/10.0 Operation: lumbar spine surgery. NIRS: INVOS system.	bSO2 >75%[$] (13)	No NIRS monitoring (30)	Neurocognitive impairment (MMSE).	None

[$]Target values were set on the basis of baseline bSO_2 measurement. [¥]only serum S100B was dosed.

Table S3. Risk of bias in studies eligible.

Trials	Random Sequence Generation	Allocation Concealment	Blinding of Participants and Personnel	Blinding of Outcome Assessment	Incomplete Outcome Data	Selective Reporting	Other Bias
**Colak et al., 2015** [1]	L	L	L	U	U	L	U
**NORMOSAT** [2]	L	L	L	L	U	L	U
**Kara et al., 2016** [3]	L	L	L	L	L	L	U
**Mohandas et al., 2013** [4]	L	L	U	U	U	L	U
**Slater et al., 2009** [5]	L	L	L	L	L	L	U
**Murkin et al., 2007** [6]	L	L	U	U	U	L	U
**Vretzakis et al., 2013** [7]	L	L	U	U	U	L	U
**PASPORT** [8]	L	L	L	U	U	L	U
**Cowie et al., 2014** [9]	L	L	L	L	U	L	U
**Ballard et al., 2012** [10]	L	L	U	U	U	U	U
**Casati et al., 2007** [11]	L	L	U	U	U	U	U
**Cox et al., 2018** [12]	L	L	U	U	L	U	U
**Deschamps et al., 2013** [13]	L	L	U	U	U	L	U
**Harilall et al., 2014** [14]	L	L	U	U	L	U	U
**Trafidło et al., 2015** [15]	L	L	U	U	U	U	U

The judgement for each of risk of bias domain is presented as (L), (U) or (H) to indicate low, unclear, or high risk of bias, respectively.

Study or Subgroup	NIRS Events	NIRS Total	SOC Events	SOC Total	Weight	Odds Ratio M-H, Random, 95% CI
Cowie et al., 2014	0	20	1	20	8.7%	0.32 [0.01, 8.26]
Murkin et al., 2007	0	100	1	100	8.9%	0.33 [0.01, 8.20]
NORMOSAT	4	102	6	99	54.8%	0.63 [0.17, 2.31]
PASPORT	1	98	2	106	15.8%	0.54 [0.05, 6.01]
Vretzakis et al., 2013	1	70	1	67	11.8%	0.96 [0.06, 15.61]
**Total (95% CI)**		**390**		**392**	**100.0%**	**0.58 [0.22, 1.50]**
Total events	6		11			

Heterogeneity: $Tau^2 = 0.00$; $Chi^2 = 0.40$, df = 4 (P = 0.98); $I^2 = 0\%$

Test for overall effect: Z = 1.13 (P = 0.26)

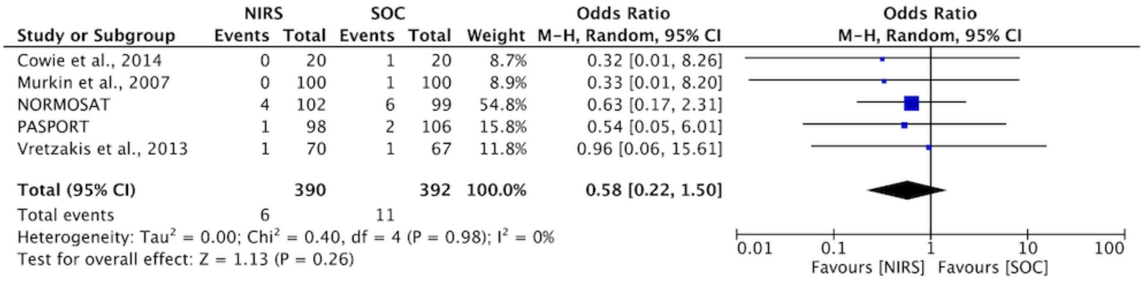

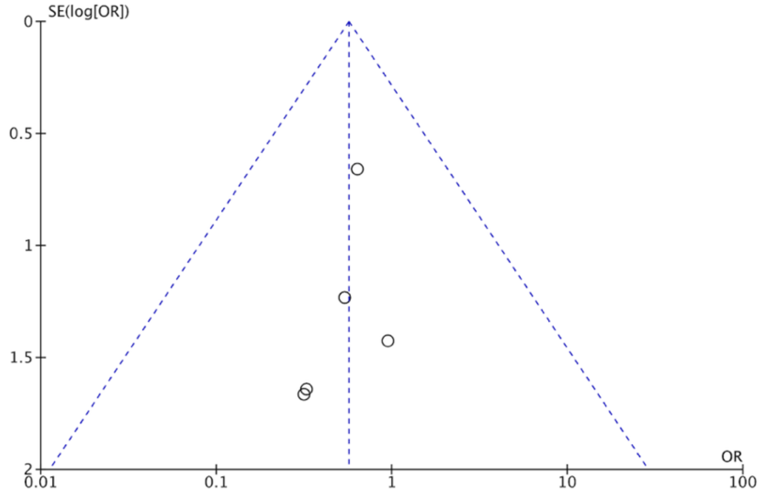

Figure S1. The effect of NIRS monitoring on post-operative death. CI, confidence interval; M-H, Mantel–Haenszel test; NIRS, near-infrared spectroscopy; NORMOSAT, NORMal cerebral Oxygen SATuration; PASPORT, PAtient-Specific cerebral oxygentation monitoring as Part Of an algorithm to Reduce Transfusion during heart valve surgery: a randomised controlled trial; SE, standard error; SOC, standard of care.

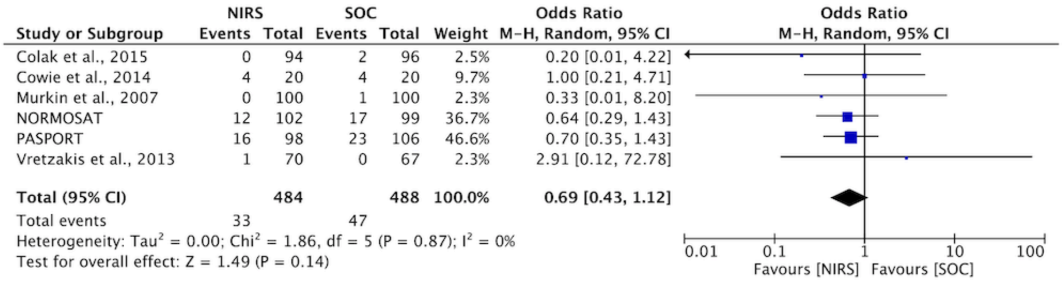

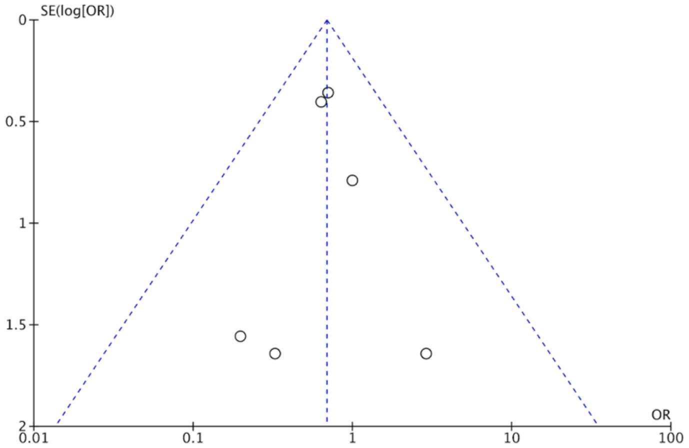

Figure S2. The effect of NIRS monitoring on post-operative AKI. AKI, acute kidney injury; CI, confidence interval; M-H, Mantel–Haenszel test; NIRS, near-infrared spectroscopy; NORMOSAT, NORMal cerebral Oxygen SATuration; PASPORT, PAtient-Specific cerebral oxygentation monitoring as Part Of an algorithm to Reduce Transfusion during heart valve surgery: a randomised controlled trial; SE, standard error; SOC, standard of care.

Study or Subgroup	NIRS Events	NIRS Total	SOC Events	SOC Total	Weight	Odds Ratio M-H, Random, 95% CI
Colak et al., 2015	1	94	4	96	14.9%	0.25 [0.03, 2.25]
Cowie et al., 2014	1	102	2	99	12.5%	0.48 [0.04, 5.38]
Murkin et al., 2007	2	100	7	100	28.6%	0.27 [0.05, 1.34]
NORMOSAT	0	20	1	20	6.9%	0.32 [0.01, 8.26]
PASPORT	2	98	2	100	18.6%	1.02 [0.14, 7.39]
Vretzakis et al., 2013	2	70	2	67	18.4%	0.96 [0.13, 6.99]
**Total (95% CI)**		**484**		**482**	**100.0%**	**0.47 [0.20, 1.10]**
Total events	8		18			

Heterogeneity: $Tau^2 = 0.00$; $Chi^2 = 1.93$, df = 5 (P = 0.86); $I^2 = 0\%$
Test for overall effect: Z = 1.74 (P = 0.08)

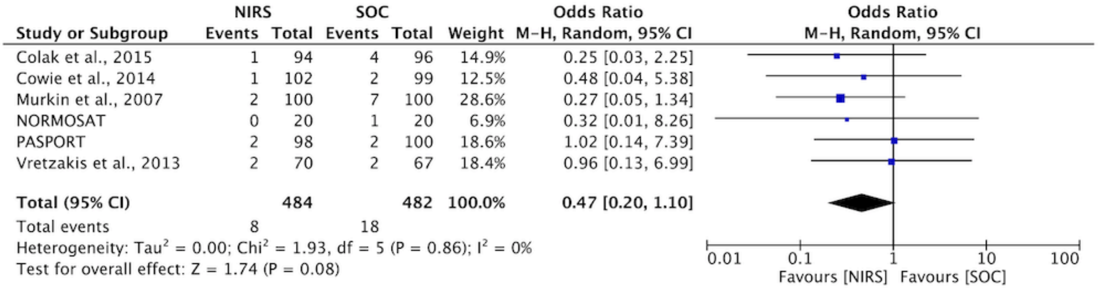

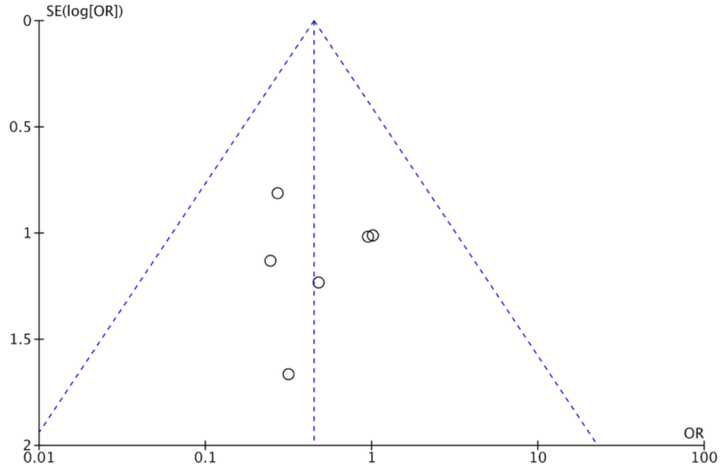

Figure S3. The effect of NIRS monitoring on MACEs. CI, confidence interval; MACE, major adverse cardiovascular event; M-H, Mantel–Haenszel test; NIRS, near-infrared spectroscopy; NORMOSAT, NORMal cerebral Oxygen SATuration; PASPORT, PAtient-Specific cerebral oxygentation monitoring as Part Of an algorithm to Reduce Transfusion during heart valve surgery: a randomised controlled trial; SE, standard error; SOC, standard of care.

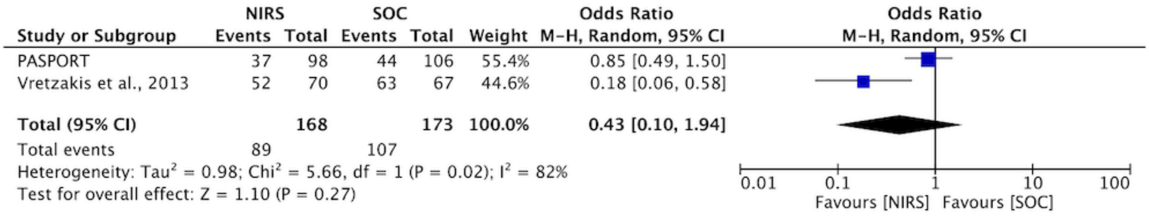

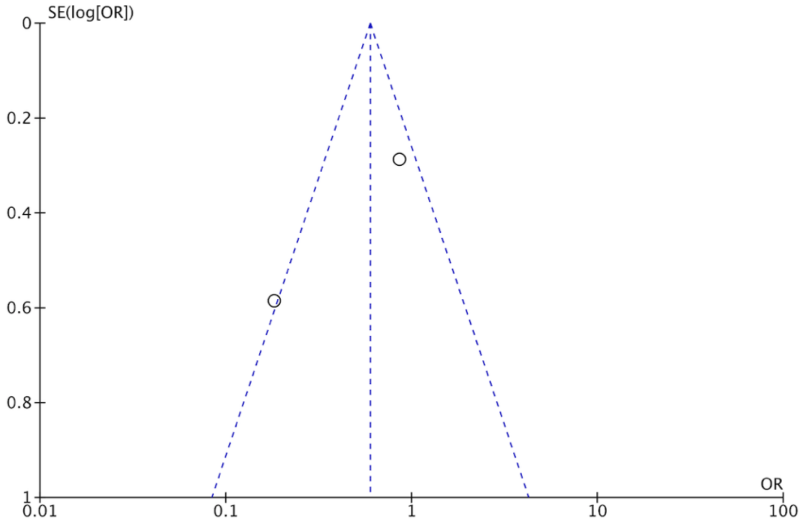

Figure S4. The effect of NIRS monitoring on bleeding/need for transfusions. CI, confidence interval; M-H, Mantel–Haenszel test; NIRS, near-infrared spectroscopy; PASPORT, PAtient-Specific cerebral oxygentation monitoring as Part Of an algorithm to Reduce Transfusion during heart valve surgery: a randomised controlled trial; SE, standard error; SOC, standard of care.

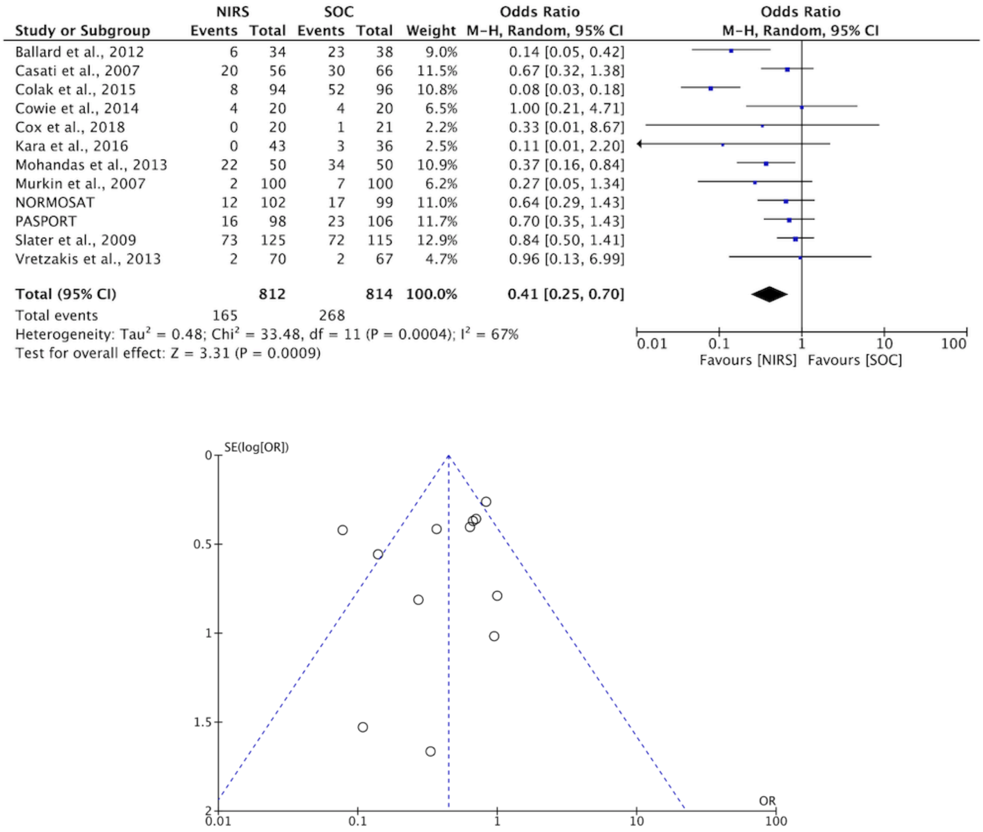

Figure S5. The effect of NIRS monitoring on morbidity combining POCD/POD, AKI and MACEs. CI, confidence interval; M-H, Mantel–Haenszel test; NIRS, near-infrared spectroscopy; NORMOSAT, NORMal cerebral Oxygen SATuration; PASPORT, PAtient-Specific cerebral oxygentation monitoring as Part Of an algorithm to Reduce Transfusion during heart valve surgery: a randomised controlled trial; SE, standard error; SOC, standard of care.

Table S4. The ranking of SUCRA assessable NIRS target values for three outcomes.

**NIRS Target Values** † **INVOS and Non-INVOS Systems.**	**SUCRA** ‡ **Outcomes: A/B/C** §
bSO_2 > 75%	0.6717/0.5011/0.7696
bSO_2 > 80%	NAs/0.8406/0.6852
bSO_2 > 70%	NAs/0.4099/0.5514
Non-drop bSO_2 > 20%	0.5623/0.3992/0.4073
SOC	0.2659/0.3492/0.0864

§ The following outcomes providing numerical data were considered: (A) peri-/post-operative death, and (B) post-operative acute kidney function deterioration and (C) cardiovascular events occurrence. † For both INVOS and non-INVOS system for monitoring, the NIRS target values presented were ranked according to probabilities for being the best, the second best, the third best, and so on $P(v = b)$, $b = 1, \ldots, a$ following Markov chain Monte Carlo methods. ‡ SUCRA for each target value v out of the a competing target values requires calculation of the a vector of the cumulative probabilities $cum_{v,b}$ to be among the b best target value, $b = 1, \ldots, a$. NAs, non-assessable; NIRS, near-infrared spectroscopy; SOC, standard or care; SUCRA, surface under the cumulative ranking area.

A

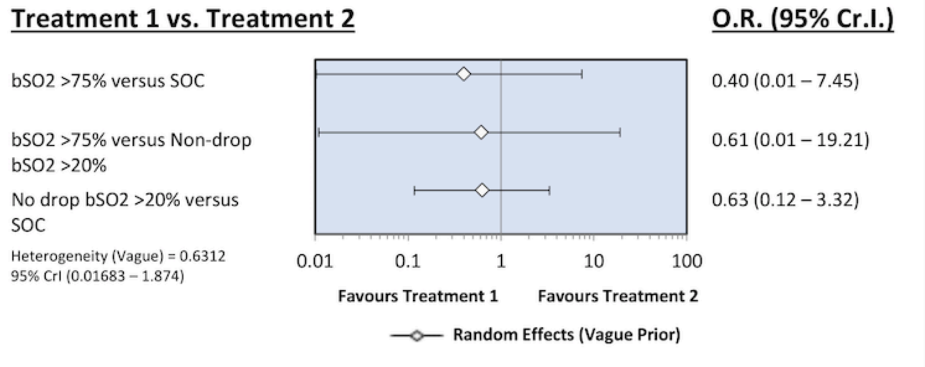
Treatment 1 vs. Treatment 2
O.R. (95% Cr.I.)
bSO2 >75% versus SOC
0.40 (0.01 – 7.45)
bSO2 >75% versus Non-drop bSO2 >20%
0.61 (0.01 – 19.21)
No drop bSO2 >20% versus SOC
0.63 (0.12 – 3.32)
Heterogeneity (Vague) = 0.6312
95% CrI (0.01683 – 1.874)
0.01
0.1
1
10
100
Favours Treatment 1
Favours Treatment 2
Random Effects (Vague Prior)

B

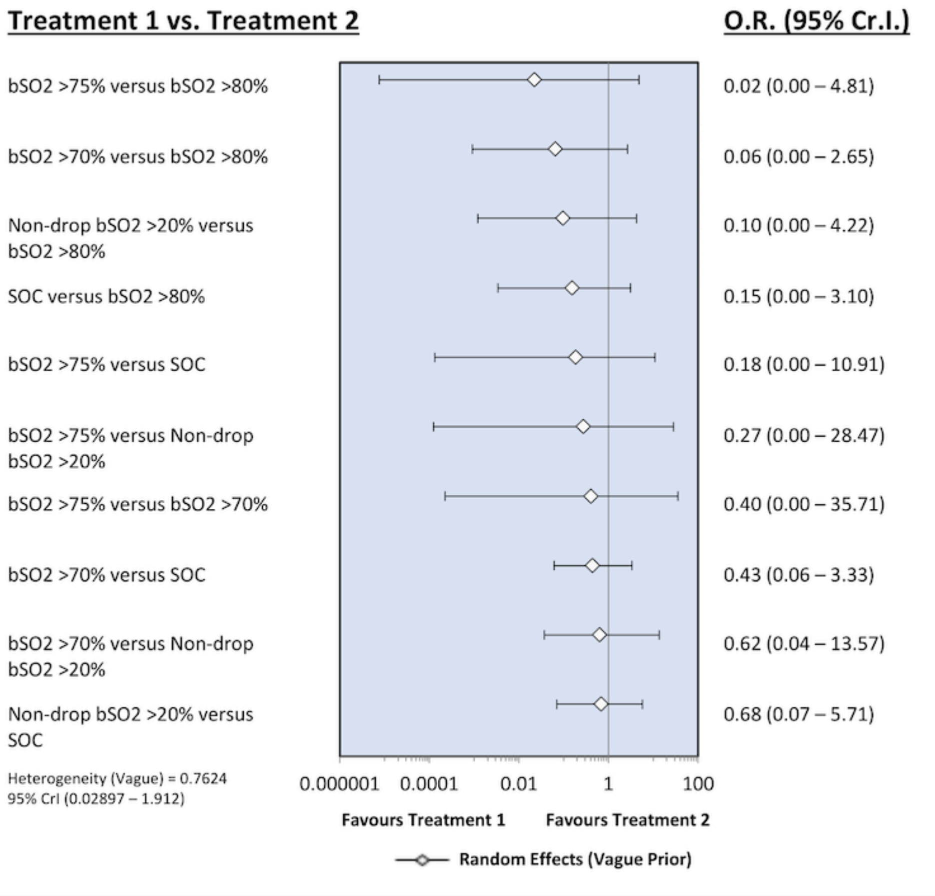
Treatment 1 vs. Treatment 2
O.R. (95% Cr.I.)
bSO2 >75% versus bSO2 >80%
0.02 (0.00 – 4.81)
bSO2 >70% versus bSO2 >80%
0.06 (0.00 – 2.65)
Non-drop bSO2 >20% versus bSO2 >80%
0.10 (0.00 – 4.22)
SOC versus bSO2 >80%
0.15 (0.00 – 3.10)
bSO2 >75% versus SOC
0.18 (0.00 – 10.91)
bSO2 >75% versus Non-drop bSO2 >20%
0.27 (0.00 – 28.47)
bSO2 >75% versus bSO2 >70%
0.40 (0.00 – 35.71)
bSO2 >70% versus SOC
0.43 (0.06 – 3.33)
bSO2 >70% versus Non-drop bSO2 >20%
0.62 (0.04 – 13.57)
Non-drop bSO2 >20% versus SOC
0.68 (0.07 – 5.71)
Heterogeneity (Vague) = 0.7624
95% CrI (0.02897 – 1.912)
0.000001
0.0001
0.01
1
100
Favours Treatment 1
Favours Treatment 2
Random Effects (Vague Prior)

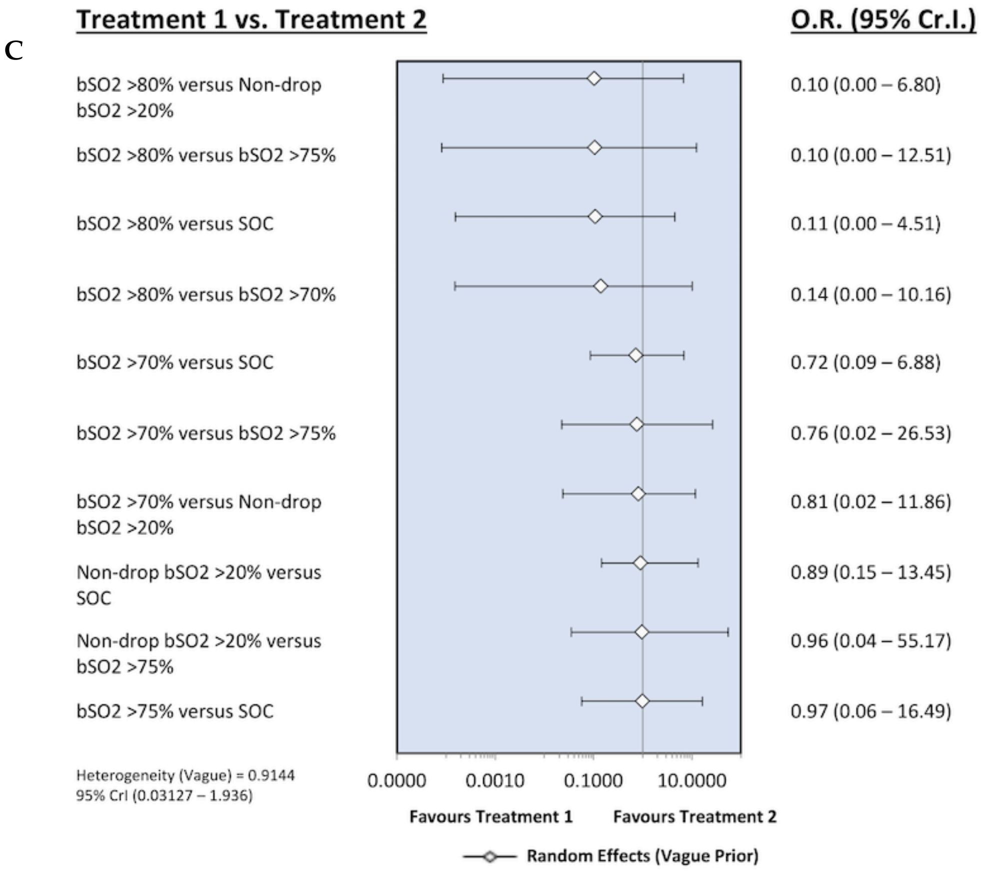

Figure S6. Network forests plot of the random effects (vague priors) for the SUCRA assessable NIRS target values corresponding to (**A**) post-operative death, (**B**) post-operative AKI, and (**C**) MACEs. CrI, credible intervals; OR, odds ratio; SOC, standard of care.

A

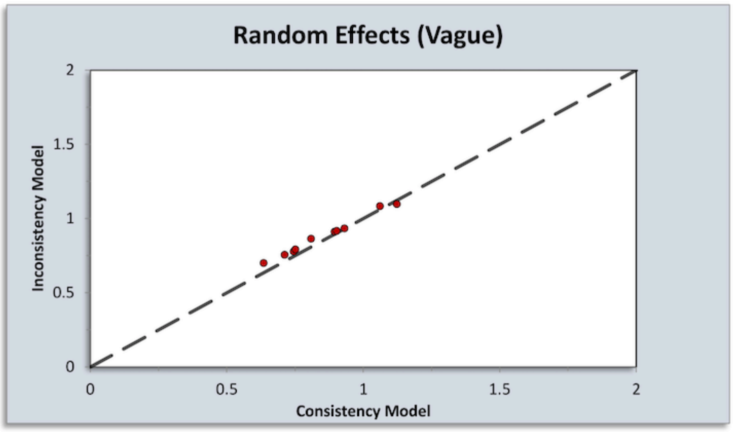

B

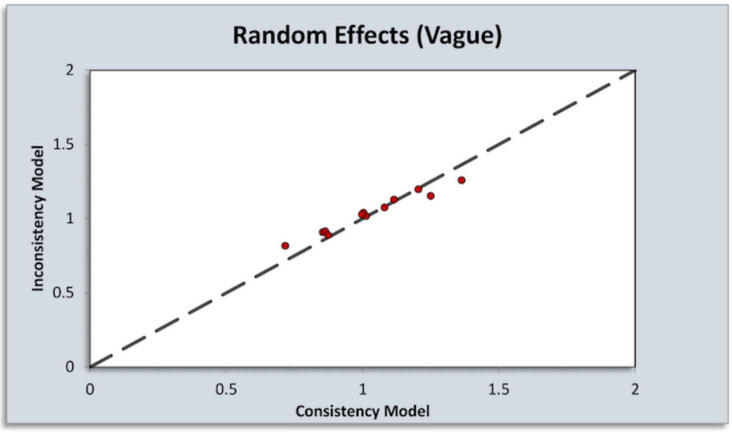

C

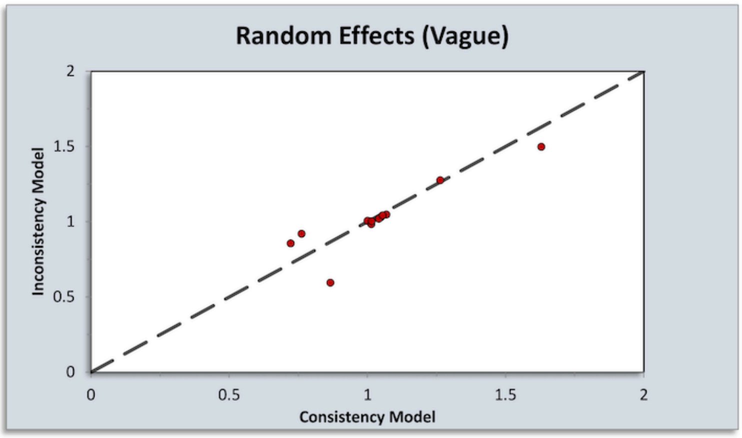

Figure S7. Inconsistency plot of the random effects for the SUCRA assessable NIRS target values corresponding to (**A**) post-operative death, (**B**) post-operative AKI, and (C) MACEs.

References

1. Colak, Z.; Borojevic, M.; Bogovic, A.; Ivancan, V.; Biocina, B.; Majeric-Kogler, V. Influence of intraoperative cerebral oximetry monitoring on neurocognitive function after coronary artery bypass surgery: A randomized, prospective study. *Eur. J. Cardiothorac. Surg.* **2015**, *47*, 447–454. https://doi.org/10.1093/ejcts/ezu193.
2. Deschamps, A.; Hall, R.; Grocott, H.; Mazer, C.D.; Choi, P.T.; Turgeon, A.F.; de Medicis, E.; Bussières, J.S.; Hudson, C.; Syed, S.; et al. Cerebral Oximetry Monitoring to Maintain Normal Cerebral Oxygen Saturation during High-risk Cardiac Surgery: A Randomized Controlled Feasibility Trial. *Anesthesiology* **2016**, *124*, 826–836. https://doi.org/10.1097/ALN.0000000000001029.
3. Kara, I.; Erkin, A.; Saclı, H.; Demirtas, M.; Percin, B.; Diler, M.S.; Kırali, K. The Effects of Near-Infrared Spectroscopy on the Neurocognitive Functions in the Patients Undergoing Coronary Artery Bypass Grafting with Asymptomatic Carotid Artery Disease: A Randomized Prospective Study. *Ann. Thorac. Cardiovasc. Surg.* **2015**, *21*, 544–550. https://doi.org/10.5761/atcs.oa.15-00118.
4. Mohandas, B.S.; Jagadeesh, A.M.; Vikram, S.B. Impact of monitoring cerebral oxygen saturation on the outcome of patients undergoing open heart surgery. *Ann. Card. Anaesth.* **2013**, *16*, 102–106. https://doi.org/10.4103/0971-9784.109740.
5. Slater, J.P.; Guarino, T.; Stack, J.; Vinod, K.; Bustami, R.T.; Brown III, J.M.; Rodriguez, A.L.; Magovern, C.J.; Zaubler, T.; Freundlich, K.; et al. Cerebral oxygen desaturation predicts cognitive decline and longer hospital stay after cardiac surgery. *Ann. Thorac. Surg.* **2009**, *87*, 36–45. https://doi.org/10.1016/j.athoracsur.2008.08.070.
6. Murkin, J.M.; Adams, S.J.; Novick, R.J.; Quantz, M.; Bainbridge, D.; Iglesias, I.; Cleland, A.; Schaefer, B.; Irwin, B.; Fox, S. Monitoring brain oxygen saturation during coronary bypass surgery: A randomized, prospective study. *Anesth. Analg.* **2007**, *104*, 51–58. https://doi.org/10.1213/01.ane.0000246814.29362.f4.
7. Vretzakis, G.; Georgopoulou, S.; Stamoulis, K.; Tassoudis, V.; Mikroulis, D.; Giannoukas, A.; Tsilimingas, N.; Karanikolas, M. Monitoring of brain oxygen saturation (INVOS) in a protocol to direct blood transfusions during cardiac surgery: A prospective randomized clinical trial. *J. Cardiothorac. Surg.* **2013**, *8*, 145. https://doi.org/10.1186/1749-8090-8-145.
8. Rogers, C.A.; Stoica, S.; Ellis, L.; Stokes, E.A.; Wordsworth, S.; Dabner, L.; Clayton, G.; Downes, R.; Nicholson, E.; Bennett, S.; et al. Randomized trial of near-infrared spectroscopy for personalized optimization of cerebral tissue oxygenation during cardiac surgery. *Br. J. Anaesth.* **2017**, *119*, 384–393. https://doi.org/10.1093/bja/aex182.
9. Cowie, D.A.; Nazareth, J.; Story, D.A. Cerebral oximetry to reduce perioperative morbidity. *Anaesth. Intensive Care* **2014**, *42*, 310–314. https://doi.org/10.1177/0310057X1404200306.
10. Ballard, C.; Jones, E.; Gauge, N.; Aarsland, D.; Nilsen, O.B.; Saxby, B.K.; Lowery, D.; Corbett, A.; Wesnes, K.; Katsaiti, E. Optimised anaesthesia to reduce post operative cognitive decline (POCD) in older patients undergoing elective surgery, a randomised controlled trial. *PLoS ONE* **2012**, *7*, e37410. https://doi.org/10.1371/journal.pone.0037410.
11. Casati, A.; Fanelli, G.; Pietropaoli, P.; Proietti, R.; Tufano, R.; Montanini, S. Monitoring cerebral oxygen saturation in elderly patients undergoing general abdominal surgery: A prospective cohort study. *Eur. J. Anaesthesiol.* **2007**, *24*, 59–65. https://doi.org/10.1017/S0265021506001025.
12. Cox, R.M.; Jamgochian, G.C.; Nicholson, K.; Wong, J.C.; Namdari, S.; Abboud, J.A. The effectiveness of cerebral oxygenation monitoring during arthroscopic shoulder surgery in the beach chair position: A randomized blinded study. *J. Shoulder Elbow Surg.* **2018**, *27*, 692–700. https://doi.org/10.1016/j.jse.2017.11.004.
13. Deschamps, A.; Lambert, J.; Couture, P.; Rochon, A.; Lebon, J.S.; Ayoub, C.; Cogan, J.; Denault, A. Reversal of decreases in cerebral saturation in high-risk cardiac surgery. *J. Cardiothorac. Vasc. Anesth.* **2013**, *27*, 1260–1266. https://doi.org/10.1053/j.jvca.2013.01.019.
14. Harilall, Y.; Adam, J.K.; Biccard, B.M.; Reddi, A. The effect of optimising cerebral tissue oxygen saturation on markers of neurological injury during coronary artery bypass graft surgery. *Heart Lung Circ.* **2014**, *23*, 68–74. https://doi.org/10.1016/j.hlc.2013.07.002.
15. Trafidło, T.; Gaszyński, T.; Gaszyński, W.; Nowakowska-Domagała, K. Intraoperative monitoring of cerebral NIRS oximetry leads to better postoperative cognitive performance: A pilot study. *Int. J. Surg.* **2015**, *16*, 23–30. https://doi.org/10.1016/j.ijsu.2015.02.009.
